# Tea consumption didn’t modify the risk of fracture: a dose–response meta-analysis of observational studies

**DOI:** 10.1186/1746-1596-9-44

**Published:** 2014-03-03

**Authors:** Bo Chen, Hai-Fei Shi, Shou-Cheng Wu

**Affiliations:** 1 Department of Hand Surgery, The First Affiliated Hospital, Zhejiang University School of Medicine, Zhejiang, China

## Abstract

**Background:**

Fractures are important causes of healthy damage and economic loss nowadays. The conclusions of observational studies on tea consumption and fracture risk are still inconsistent. The objective of this meta-analysis is to determine the effect of tea drinking on the risk of fractures.

**Methods:**

A comprehensive literature search was conducted in PubMed, Embase and reference lists of the relevant articles. Observational studies that reported an estimate of the association between tea drinking and incidence of fractures were included. A meta-analysis was conducted by the STATA software.

**Results:**

A total of 9 studies involving 147,950 individuals that examined the association between tea consumption and risk of fractures were included in this meta-analysis. The odds risks (ORs) with 95% confidence intervals (CIs) were pooled using a random-effects model. The pooled OR of 9 observational studies for the tea consumption on risk of fracture was 0.89 (95% CI, 0.78-1.04). In the subgroup analyses, no significant association was detected in neither cohort studies (n = 3; OR, 0.97; 95% CI, 0.89-1.06) nor case–control studies (n = 6; OR, 0.91; 95% CI, 0.70-1.19), respectively. No significant association was detected in the dose–response meta-analysis.

**Conclusions:**

Tea consumption might not be associated with the risk of fractures. The following large-sample and well-designed studies are required to confirm the existing conclusions.

**Virtual slides:**

The virtual slide(s) for this article can be found here: http://www.diagnosticpathology.diagnomx.eu/vs/5309904231178427.

## Introduction

Fractures are important causes of healthy damage and economic loss in the whole world. Several risk factors have been detected including the lower body mass density (BMD) [1] and the increased body mass index (BMI). Besides, the tobacco exposure [2], coffee intake [3], calcium and vitamin D intake [4] and history of fractures are also considered to be associated with the risk of fractures. The realization of the relationship between these modifiable factors and incidence of fractures would provide more effective strategy for the fracture prevention in the future.

Tea is the most popular beverage in the world. Several kinds of antioxidants, including flavonoid, catechin, thearubigin and theaflavin, exist in the tea. Nowadays, the tea drinking behave is considered to be associated with the development and progression of different kinds of diseases. Tea consumption is reported to be a protective factor of the cardiovascular diseases [5], Parkinson’s disease [6] and several kinds of cancers [7,8]. Whatsmore, habitual tea drinking is reported to have a positive effect on the BMD [9,10], which may thus prevent the incidence of fracture (osteoporotic fracture, in particular). However, tea is a beverage which contains caffeine as well. It was reported that the caffeine could decrease the BMD and subsequently increase the risk of fractures [11]. The contradictory effects of tea drinking make its role on the risk of fractures indistinct. Several epidemiological studies have researched the relationship between tea consumption and incidence of fracture; however, no accordant conclusion was obtained by now [10,12]. Meta-analysis is a useful statistical tool to pool the relevant studies together and gain a more powerful conclusion. For instance, meta-analysis was used to detect the risk factors of lung cancer [13], breast cancer [14], type 2 diabetes [15] and thyroid lesions [16]. The purposes of this study were: firstly, quantify the effect of tea consumption on the incidence of fractures by meta-analyzing the existing observational studies and provide quantitative and high-level evidence and secondly, provide some suggestions for the following advanced studies.

## Methods

### Search strategy and inclusion criteria

This current meta-analysis was conducted according to the PRISMA guidelines and MOOSE guidelines [17,18]. We searched two databases (Pubmed and EMBASE) with the key words “tea”, “green tea”, “black tea”, “flavonoid”, “catechin”, “thearubigin”, “polyphenol”, “theaflavin” in combine with “fracture” through July 8, 2013. No language or any other restrictions were set in the search strategy. The additional articles were obtained through consulting the reference lists of the relevant studies. When different articles from the same dataset were obtained, only the most recent study with available data was included in the meta-analysis. The contact with the corresponding author of certain article was conducted when more data was required.

A study would be included in this meta-analysis when it met the following inclusion criteria: [1] the association between tea consumption and risk of fractures was evaluated; [2] a cohort or case–control study design was obtained; [3] the value of relative risk (RR), odds ratio (OR) with 95% confidence intervals (CI) or enough data to calculate them were reported.

### Data extraction and assessment of study quality

The data of each included article was extracted by two reviewers (Bo Chen and Hai-Fei Shi ) independently and any discrepancy was checked again and resolved through discussion. The following data was extracted from each article: the name of the first author, publishing year, study design, study site, age and gender of participants, type and amount of cases, adjustments of the confounding factors, and the OR/RR value with 95% CI.

The study quality was assessed by two reviewers back to back and any disagreement was discussed with the third reviewer (Shou-Cheng Wu). All the included studies are cohort or case–control studies. Considering the observational study design of the included studies, the Newcastle-Ottawa Scale (NOS) was obtained to assess the methodological quality of the included studies [19]. It assessed the selection, comparability and exposure of a case–control study, while the selection, comparability and outcome of a cohort study. A maximum of 9 stars was scored for a study and the study with over 6 stars would be regarded as relative high quality.

### Statistical methods for the meta-analysis

Both OR and RR were reported in the included studies and the OR was obtained to approximate RR in this meta-analysis. When both crude and adjusted OR/RR values were offered in the article, only the adjusted ones were adopted for the meta-analysis. A random-effects model was obtained to estimate the pooled effects, thus it would provide a more conservative conclusion. The effects of tea drinking on the incidence of fractures were measured with the OR with 95% CI. The heterogeneity among the included studies was detected by both *χ*^*2*^ and *I*^*2*^ tests. When *P* for the heterogeneity < 0.1 and *I*^*2*^ > 50%, the heterogeneity would be considered statistically significant. The source of the statistically significant heterogeneity was explored by both removing the included studies one by one and conducting the subgroup analysis. A cumulative meta-analysis was conducted as well through sorting all the studies by the publishing year.

We examined a potential nonlinear dose–response relationship between tea consumption and fracture risk using restricted cubic splines with three knots at percentiles 25%, 50%, and 75% of the distribution. A two-stage random-effects dose–response meta-analysis tested was adopted for the detection of the potential nonlinear relation. A P value for nonlinearity was calculated by testing the null hypothesis that the coefficient of the second spline is equal to 0 [20,21].

The sensitivity analyses were conducted to detect the robustness of the conclusions by two independent methods. Firstly, we conducted a one-way sensitivity analysis in which the included study was excluded one by one. Secondly, we excluded the studies with a relative lower methodological quality and assess the effect of tea consumption and risk of fracture. The potential publication bias was estimated by visually evaluation of funnel plot, Bgger’s test and Egger’s test concurrently [22,23]. The Stata software package (version 11.0; Stata Corp., College Station, TX) was obtained to conduct the meta-analysis.

## Results

### Identification and selection of studies

**Figure 1:**
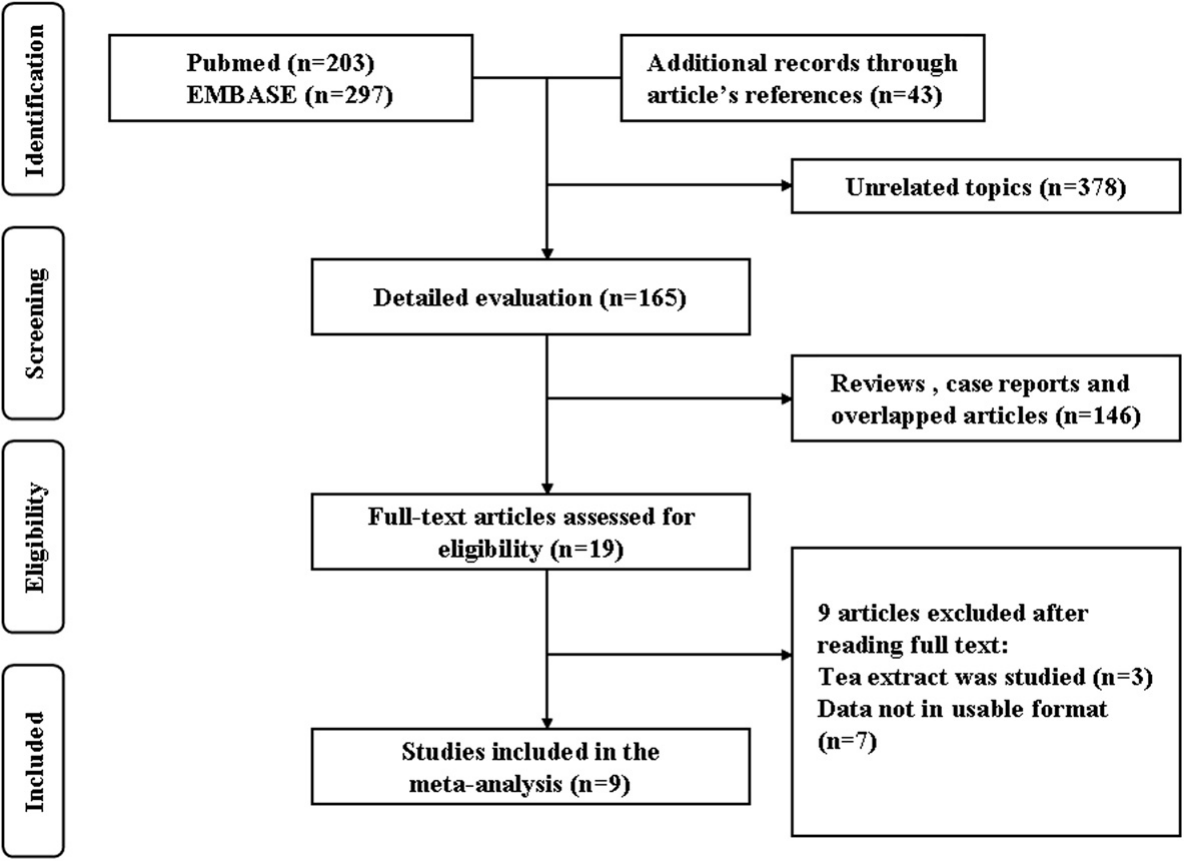
**Flow diagram showing the identification of relevant studies in the meta-analysis.** A total of 500 articles (203 from Pubmed and 297 from EMBASE) were identified from the electronic database searching. Besides, 43 more records were identified through consulting the citations of the relevant reviews and articles. A total of 165 records were detailed evaluated and19 full-text were assessed for eligibility after removing 146 reviews, case reports and overlapped articles. Subsequently 3 articles in which tea extract was studied and 7 ones in which the data not in usable format were excluding from the inclusion and in final, a total of 9 articles were included for the quantitative synthesis.

A total of 500 articles (203 from Pubmed and 297 from EMBASE) were identified from the electronic database searching. Besides, 43 more records were identified through consulting the reference lists of the relevant reviews and articles. After excluding 378 articles with unrelated topics, a total of 165 records were detailed evaluated. Among the 165 articles, 19 full-texts were assessed for eligibility after removing 146 articles (reviews, case reports and overlapped articles). Subsequently 3 articles in which tea extract was studied and 7 ones in which the data not in usable format were excluding from the inclusion and in final, a total of 9 studies were included for the quantitative synthesis [24-33] (Figure 1).

### Study characteristics and quality

A total of 17,965 cases and 147,950 individuals in 9 observational studies were indemnified in this current meta-analysis. All the detailed characteristics of each included study were presented in Table 1. The included studies were published between 1992 and 2013. A total of 3 cohort studies and 6 case–control studies were identified in this current meta-analysis. Among all the included studies, 4 studies were in European, 2 in Americas and 3 in Asia. The age, gender distribution, categories of tea consumption, and adjustments of confounding factors were demonstrated in Table 1.

**Table 1:** Baseline of the included studies

**Author**	**Year**	**Study design**	**Site**	**Age (year)**	**Gender**	**Type and No. of fractures**	**No. of participants**	**Categories of tea consumption**	**Adjustment**
Kreiger N [17]	1992	Case–control	Canada	50-84	Female	Hip fracture 102, wrist fracture 154	533	2 categories, <3 cup/day, ≥ 3 cup/day	Age, the Queteletindex, ovariectomy, estrogens replacement, cigarette smoking
Johnell O [18]	1995	Case–control	Southern Europe	> 50	Female	Hip fracture 2086	5,618	4 categories, never, 1–2 cups/day, 3 cups/day, ≥ 4 cups/day	Age, center and BMI
Tavani A [19]	1995	Case–control	Italy	19-74	Female	Hip fracture 279	1,340	2 categories, no drinking, drinking	Age, education, body mass index, smoking status, total alcohol consumption, calcium intake, menopausal status, and estrogen replacement therapy use
Chen Z [20]	2003	Cohort	USA	50-79	Female	Hip fractures 386, forearm/wrist fractures 1,809, other fractures 8,332	91,465	4 categories, <1 cup/day 1 cup/day 2–3 cups/day ≥ 4 cups/day	Age, BMI, ethnicity, hormone replacement therapy use, smoking, years since menopause, fracture history, osteoporosis drug use, walking, soy milk consumption, coffee drinking, and depression
Hallström H [21]	2010	Case–control	India	65.2 ± 15.1	M:F 86:114	Hip fracture, 100	200	2 categories, ≤ 1 cup/day >1 cup/day	Age, BMI, agility, eat pander, eat fish, active persons, take calcium supplements
Jha RM [22]	1999	Case–control	Southern Europe	50-100	Male	Hip fracture 730	1,862	4 categories, never, sometimes, 1–2 cups/day, ≥ 3 cups/day	Age, center and BMI
Kanis J [23]	2013	Case–control	China	71 ± 7	M:F 148:433	Hip fracture femoral neck fractures 396, intertrochanteric fractures 185	581	2 categories, No drinking, drinking	NA
Zeng FF [24]	2011	Cohort	China	>90	M:F 226:467	Osteoporotic fracture 72	703	2 categories, Former drinking, current drinking	Age, gender, sleep habits educational levels, religion habits and temperament
Tomata Y [25]	2012	Cohort	Japan	>65	M:F 6176:7812	Hip fracture 55	13,988	4 categories, 1 cup/day 1–2 cups/day	NA
								3–4 cups/day	
								5 cups/day	

M: male; F: female; BMI: body mass index; NA: not available.

The NOS were obtained to assess the selection, comparability and exposure of the case–control study, while the selection, comparability and outcome of the cohort study. All the scores of each part in the evaluation of all the studies were displayed in Table 2. Eight in nine studies were in relative high quality (over 6 stars) and the mean NOS score was 6.90 stars (standard deviation: 1.37).

**Table 2:** **Quality assessment of included studies **^**a**^

		**Quality assessment criteria**			
**Author**	**Study design**	**Selection**	**Comparability**	**Outcome/exposure**	**Overall quality**
Kreiger N [17]	Case–control	***	**	***	8
Johnell O [18]	Case–control	***	*	**	6
Tavani A [19]	Case–control	***	*	**	7
Chen Z [20]	Cohort	***	*	**	6
Hallström H [21]	Cohort	****	**	***	9
Jha RM [22]	Case–control	**	**	*	5
Kanis J [23]	Case–control	***	**	***	8
Zeng FF [24]	Case–control	***	**	**	7
Tomata Y [25]	Cohort	***	***	**	8

^a^The study quality is evaluated by Downs and Black score and Newcastle-Ottowa Scale (NOS). The NOS were obtained to assess the selection, comparability and exposure of the case–control study, while the selection, comparability and outcome for the cohort study.

*: one point; **: two points; ***: three points.

### Tea consumption and risk of fractures

**Figure 2:**
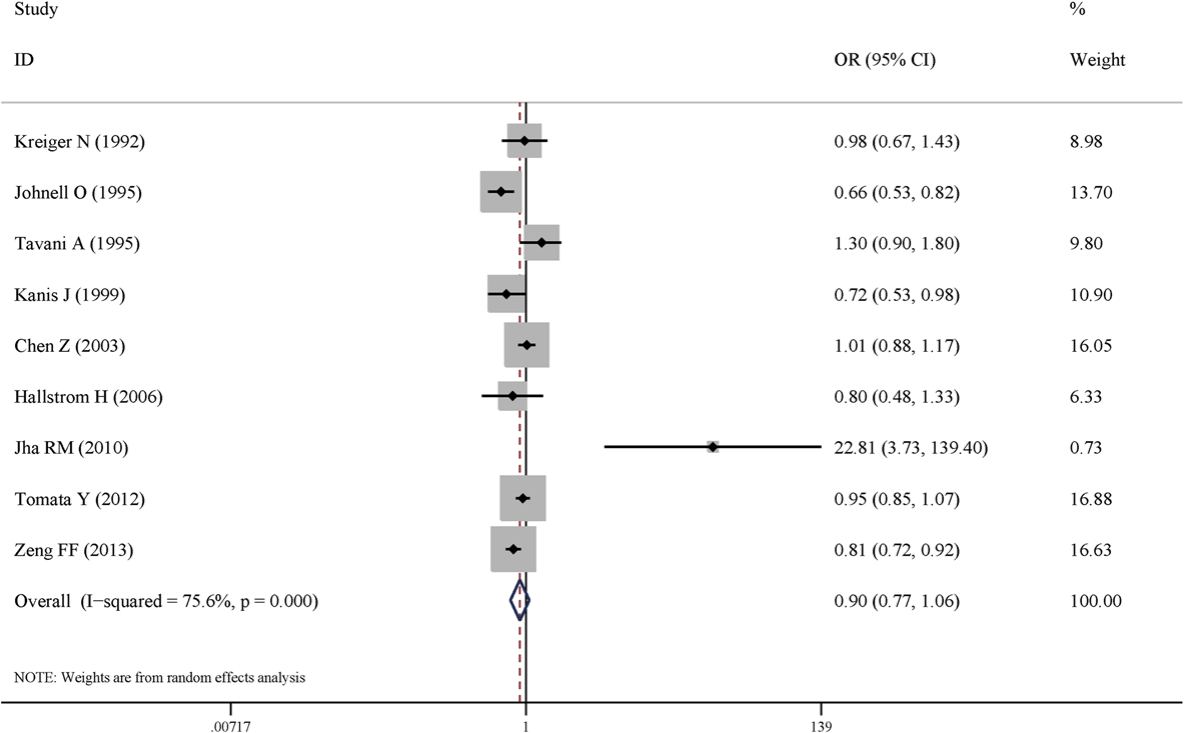
**Forest plot of risk estimates of the association between tea drinking and risk of fracture.** In a random-effects meta-analysis, tea intake was not associated the incidence of fracture (OR, 0.89; 95% CI, 0.78-1.04). A significant heterogeneity was observed when all the 9 studies were pooled (*I*^*2*^, 73.6%; *P* < 0.001).

Figure 2 showed the pooled estimation of the tea consumption and risk of fractures in a random-effects model. In this meta-analysis, tea intake was not associated the incidence of fractures (OR, 0.89; 95% CI, 0.78-1.04). No significant association was detected in neither cohort studies (n = 3; OR, 0.97; 95% CI, 0.89-1.06) nor case–control studies (n = 6; OR, 0.91; 95% CI, 0.70-1.19). When the geographical distributions of the included studies were considered, the studies that conducted in the Europe (n = 4; OR, 0.827; 95% CI, 0.61-1.13), the Americas (n = 2; OR, 1.01; 95% CI, 0.88-1.1) and the Asia (n = 3; OR, 0.921; 95% CI, 0.68-1.25) showed no statistically significant results. In the two sexual subgroups, no significant association was in neither male group (n = 2; OR, 0.818; 95% CI, 0.62-1.09) nor female group (n = 6, OR, 0.961; 95% CI, 0.77-1.20). When stratified by the fracture types, no significant association between tea consumption and hip fracture (n = 8; OR, 0.897; 95% CI, 0.74-1.09) nor humeral fracture (n = 2; OR, 0.943; 95% CI, 0.71-1.25) was detected. All the results of the subgroup analysis were presented in Table 3.

**Table 3:** Subgroup analysis of tea consumption and fracture risk with combined OR

	**Subgroups**	**No. of studies**	**Summary effect**		**Heterogeneity**		
			**OR**	**(95% CI)**	***p *Value**	***I***^***2***^**,%**	***p *Value**
Study design	Cohort	3	0.97	0.89-1.06	0.486	1.05	0.591
	Case–control	6	0.91	0.70-1.19	0.499	80.1	<0.001
Site	Europe	4	0.827	0.61-1.13	0.232	**72.4**	**0.012**
	Americas	2	1.01	0.88-1.1	0.52	0	0.857
	Asia	3	0.921	0.68-1.25	1.41	**82.2**	**0.001**
Gender	Male	2	0.818	0.62-1.09	0.168	29.9	0.232
	Female	6	0.961	0.77-1.20	0.726	**70**	**0.005**
Fracture type	Hip fracture	8	0.897	0.74-1.09	0.276	**75.8**	**<0.001**
	Humeral fracture	2	0.943	0.71-1.25	0.679	0	0.889

OR: odds ratio.

The results with statistical significance were marked in bold.

**Figure 3:**
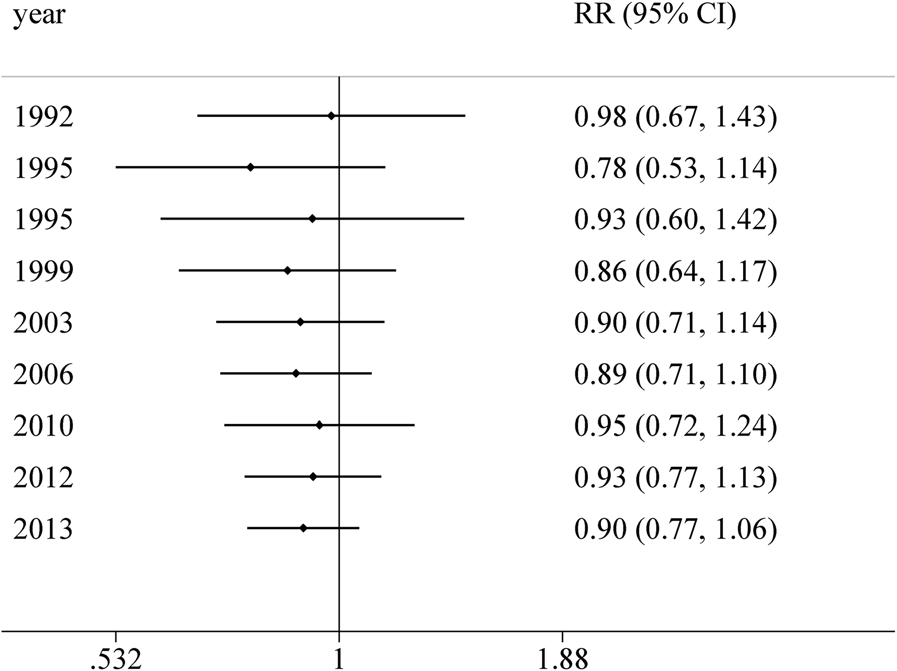
**Forest plot of the cumulative meta-analysis.** From 1992 to 2013, the tea consumption was inversely associated with the risk of fracture, however, the association was not statistical significant constantly.

A cumulative meta-analysis was conducted as well. The cumulative effect was estimated after sorting the included studies by the publishing date. Figure 3 demonstrated the forest plot of the cumulative effect of tea intake and risk of fractures. It could be detected that, from 1992 to 2013, the tea consumption was inversely associated with the risk of fractures; however, the association was not statistically significant constantly.

### Heterogeneity and sensitivity analysis

The heterogeneity was significant when all the 9 studies were pooled in the meta-analysis (*I*^*2*^, 73.6%; *P* < 0.001). We tried to explore the source by excluding the included studies one by one and re-count the heterogeneity; however, no single article could explain the source of the heterogeneity. The subgroup analysis by study design, study site, gender and fracture type demonstrated no satisfactory results in exploring the source of heterogeneity.

A one-way sensitivity analysis was conducted and Figure 3 showed the results. No significant influence was detected after any single article excluded from the meta-analysis. Besides, we just included the articles with a relative high quality (over 6 stars NOS score in the meta-analysis); however, no significant association was detected neither (OR, 0.88; 95% CI, 0.78-1.00). A significant heterogeneity should be noted as well (*I*^*2*^ = 65.9%, *P* = 0.005).

### Dose–response meta-analysis

**Figure 4:**
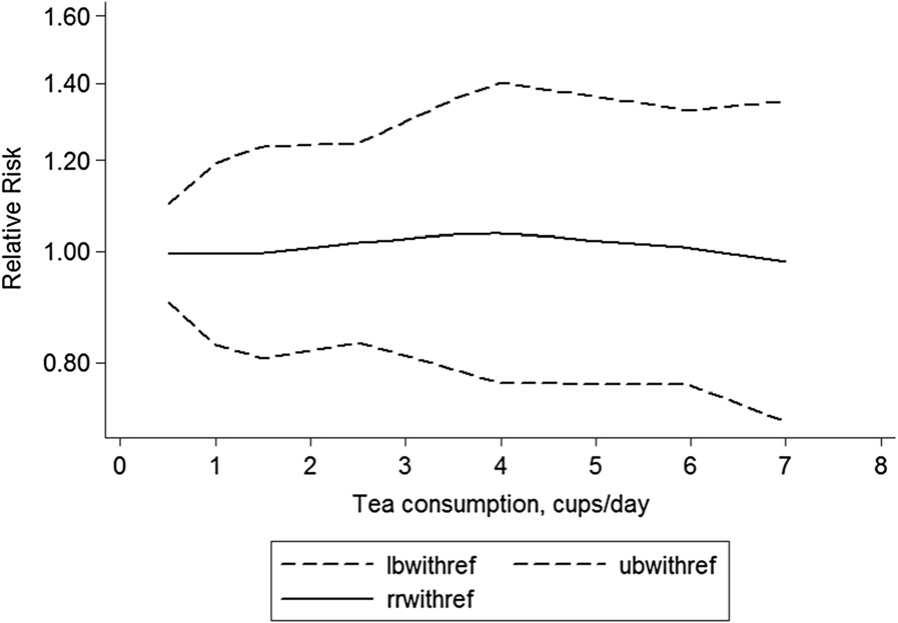
Dose–response relationship between tea intake and fracture risk.

Then, we assessed the dose–response relationship between tea consumption and the risk of fracture progression. We found no obvious evidence of statistical significance from linearity (P = 0.983). Figure 4 demonstrated the dose–response relationship between tea consumption and risk of fracture.

Both Begg’s graph and Egger’s test were obtained to detect the potential publishing bias. No significant publication bias was found in this current meta-analysis (funnel plot, symmetrical; Begg’s test, *P* = 0.474; Egger’s test, *P* = 0.627).

## Discussion

In this current meta-analysis, a total of 9 studies with 147,950 individuals were included. The quantitative synthesis of these observational studies demonstrated that tea consumption showed neither protective nor harmful effect on the incidence of fractures. Meanwhile, the subgroup analysis by the study designs, study sites, genders and fracture types displayed no statistical significant results. The cumulative meta-analysis showed that an inverse but not significant association between tea intake and risk of fractures since 1992. No significant association was detected in the dose–response meta-analysis.

Fractures are producing more and more physical ills and economic loss in the whole world. The studies on the detection of the risk factors of the fractures would provide us better knowledge on the prevention of the incidence of fractures. Several lifestyles and dietary factors, such as the tobacco exposure, coffee intake, calcium and vitamin D intake, were associated with the risk of fracture as mentioned above. Tea is regarded as the most frequently consumed beverage and the protective or harmful effects on the health are substantial public issues. As a modifiable factor, the understanding of the relationship between tea drinking and risk of fractures would provide potentially effective preventative measures of the fractures.

The association between tea consumption and BMD has been studied in several studies. However, PA collected the physical and lifestyle data from 62 postmenopausal women and the intake of tea was reported to be associated with increased BMD measurements [34]. Hegarty VM et al. compared the BMD measurements of 1,134 tea drinkers and 122 non-tea drinkers; and higher BMD was detected in the tea drinkers [35]. However, as a beverage that contains caffeine, tea drinking was also considered to be risk factors of bone loss. In animal models, high doses of caffeine could induce calcium loss and influence the normal development of bone [36]. Nowadays tea, regarded as a beverage with multiple nutrients and caffeine, is a positive factors in the bone health [37].

Several epidemiological studies were also conducted to study the association and risk of fractures directly. A cross-sectional study in Sichuan China observed the association between habits of smoking, alcohol consumption, tea consumption and exercise and osteoporotic fracture among very old people. Among all the lifestyles, tea consumption was reported to be unrelated with the incidence of osteoporotic fracture [32]. Besides, the observational studies, including cohort and case–control studies, were conducted as well to detect the etiological effect of tea consumption on the incidence of fractures. In this current meta-analysis, we studied the association between tea drinking and risk of fractures. Through pooling the published observational studies together, we found that tea consumption was not associated with the risk of fractures. This result was similar with the most included studies. Among all the included studies, only a case–control study demonstrated that tea drinking was a risk factor of the incidence of fracture [29]. Through the comparison of 100 cases and 100 age and sex matched controls, tea drinkers was reported to have a higher risk of hip fractures (OR, 22.8; 95% CI, 3.73-139.43). The ethnic and regional differences might explain part of the result differences. However, the relative smaller amount of participants would result in more unsteadiness of the result. Contrarily, three case–control studies demonstrated that tea consumption produced a protective effect on the incidence of fracture [25,30,31]. However, these studies might not be powerful enough to influence the general conclusion in this meta-analysis.

In the subgroup analysis by the study designs, no association was detected between tea consumption and risk of fractures in cohort study group and case–control study group. The cohort study design could avoid the potential recall bias and would provide more credible conclusions. In our meta-analysis, the results of the subgroups from both study designs were accordant and thus the conclusion was quite substantial. Besides, the sensitivity analysis when the studies with high quality were included showed a slight but not significant result (OR, 0.88; 95% CI, 0.78-1.00). Moreover, the cumulative meta-analysis showed that the association between tea drinking and incidence of fracture was not statistically significant; however, a potential trend that indicated the protective effect from tea consumption couldn’t be ignored.

The data about the tea or tea extract on the bone metabolism is abundant. The tea contains several kinds of antioxidants, including flavan-3-ols, flavanols and flavones, and these polyphenol produced multiple effects on the bone metabolism. At a low concentration, polyphenol would increase osteoblast proliferation, while at a high concentration, polyphenol would inhibit its proliferation inversely [37]. Oka Y et al. reported that polyphenols, particularly epigallocatechin-3-gallate (EGCG), which were extracted from the green tea, could inhibit MMPs expression and activity. Meanwhile the tea polyphenols could inhibit osteoclast formation and differentiation in vitro [38]. Choi EM reported that treatment with catechin increased alkaline phosphatase activity which could indicate the activity of osteoblastic and reduced osteoblastic apoptosis induced by the tumor necrosis factor (TNF) [39]. Oxidative stress is regarded as a pivotal physiopathological process in the age-related bone loss [40]. Das AS et al. showed that the black tea extract would protect the mononuclear cells from the oxidative stress and thus slowed the progression of bone loss [41]. Apart from the polyphenol, the other contents in tea, such as fluorine and caffeine, also play important roles in the bone health [37].

To our best knowledge, this is the first meta-analysis involving the relationship between tea consumption and risk of fracture. There are some strengths of this work. Firstly, a comprehensive literature search and advanced consulting the relevant references make available included in this meta-analysis. Secondly, the included studies are mainly of high quality and the sensitivity analysis showed that the results are quite tough, and thus the conclusions are credible.

However, there are some limitations could not be ignored in this study. Firstly, most of the studies followed a case–control study design, and therefore there were recall and selection bias which are inherent to retrospective studies. Even through the subgroup analyses by the study designs were conducted, the efficiency was limited by the absence of enough cohort studies. Secondly, tea is mainly grouped into green tea and black tea. The functional contents and effects were not identical for there two tea subtypes and thus the potential bias should be noted. The following epidemiological might need to take this into consideration.

## Conclusion

Tea consumption might not be associated with the risk of fracture. However, these conclusions should be considered with cautions because the inherent limitations in this meta-analysis. More large-sample and well-designed studies are required to confirm the existing conclusions or detect the covered significant association in this meta-analysis.

## Competing interests

The authors declare that they have no competing interests.

## Authors’ contribution

BC and SCW provided the conduction of the whole project, HFS and SCW performed the meta-analysis, BC drafted the manuscript; BC, HFS and SCW contributed to revise the manuscript. All authors read and approved the final manuscript.
